# Dynamic contrast-enhanced magnetic resonance imaging in denervated skeletal muscle: Experimental study in rabbits

**DOI:** 10.1371/journal.pone.0215069

**Published:** 2019-04-05

**Authors:** Liang Qi, Lei Xu, Wen-Tao Wang, Yu-Dong Zhang, Rui Zhang, Yue-Fen Zou, Hai-Bin Shi

**Affiliations:** 1 Department of Radiology, The First Affiliated Hospital of Nanjing Medical University, Nanjing, PR China; 2 Department of Neurosurgery, Nanjing Children’s Hospital, Nanjing, PR China; McLean Hospital, UNITED STATES

## Abstract

**Purpose:**

To investigate the value of dynamic contrast-enhanced (DCE) magnetic resonance imaging (MRI) for evaluating denervated skeletal muscle in rabbits.

**Materials and methods:**

24 male rabbits were randomly divided into an irreversible neurotmesis group and a control group. In the experimental group, the sciatic nerves of rabbits were transected for irreversible neurotmesis model. A sham operation was performed in the control group. MRI of rabbit lower legs was performed before nerve surgery and 1 day, 3 days, 5 days, 1 week, 2 weeks, 3 weeks, 4 weeks, 6 weeks, 8 weeks, 10 weeks, and 12 weeks after surgery.

**Results:**

Signal intensity changes were seen in the left gastrocnemius muscle on the T2-weighted images. DCE-MRI derived parameters (K^trans^, K_ep_, and V_p_) were measured in vivo. In the irreversible neurotmesis group, T2-weighted images showed increased signal intensity in the left gastrocnemius muscle. K^trans^, V_p_ values changes occur as early as 1 day after denervation, and increased gradually until 4 weeks after surgery. There are significant increases in both K^trans^ and V_p_ values compared with those in the control group after surgery (*P* < 0.05). K_ep_ values show no significant difference between the irreversible neurotmesis group and the control group.

**Conclusion:**

DCE-MRI hold the promise of an early and sensitive diagnosis of denervated skeletal muscle.

## Introduction

Peripheral nerve injury leads to morphologic and metabolic changes in the target denervated skeletal muscles. Electromyography (EMG) is useful for the diagnosis of denervated muscles [1]. However, EMG sometimes presents some difficulties in the detection of denervated skeletal muscle because it is both invasive and dependent on the skill of the examiner, and it is difficult to obtain information that is useful, objective, and reproducible with EMG in the deep muscles or small intramuscular areas.

Conventional magnetic resonance imaging (MRI) has proved to be useful in the diagnosis of denervated skeletal muscle after peripheral nerve injury [2–5]. Denervated skeletal muscle usually show high signal intensity on T2-weighted MR images and normal signal intensity on T1-weighted MR images. Numerous previous articles have corroborated these findings [2, 3, 5–8].

Recently, there have been a few of investigations of functional MR imaging in the evaluation of denervated skeletal muscle [9–11]. Some previous studies found that acute and subacute denervated skeletal muscles showed increased T2, apparent diffusion coefficient (ADC) and decreased fractional anisotropy on T2 mapping, diffusion-weighted imaging (DWI), and diffusion tensor imaging (DTI), respectively [12–18]. Another functional MR imaging, dynamic contrast-enhanced (DCE) T1-weighted MR perfusion imaging, is also available. With the recent improvements in functional MR imaging, tissue perfusion assessment with DCE-MRI is routinely performed in neurology, oncology and cardiology as well as, more recently, in the imaging of ischemic skeletal muscle [19–21]. To our knowledge, few articles have used this technique to assess the denervated skeletal muscle after peripheral nerve injury. Therefore, the purpose of our study was to determine the value of DCE-MRI for evaluating the early state of denervated skeletal muscle in nerve injury models in rabbits.

## Materials and methods

### Experimental model

This study was carried out in strict accordance with the recommendations in the Guide for the Care and Use of Laboratory Animals of the National Institutes of Health. The protocol was approved by the Committee on the Ethics of Animal Experiments of The First Affiliated Hospital of Nanjing Medical university (Protocol Number: 25783). All surgery was performed under sodium pentobarbital anesthesia, and all efforts were made to minimize suffering. The animals were held in separate cages in a clean environment with proper temperature and humidity. The rabbits were subjected to 12h light /dark cycle and allowed to have food and water ad libitum. Viability and behavior were recorded every day, and body weight was recorded twice weekly. If the rabbits presented with signs of suffering (pain, weakening) or loss of more than 15% of baseline weight, the animals were humanely killed considered. We used a total of 24 male New White rabbits, each of which weighted approximately 3kg. The rabbits was numbered from 1 to 24 according to the body weight, using the random number table, the animals were divided into an irreversible neurotmesis group (group A) and a control group (group B), there were 12 rabbits in each group. The rabbits were anesthetized with 30 mgkg^−1^of pentobarbital sodium (Nembutal; Bayer, Leverkusen, Germany) injected into the external ear vein. In the group A, a 3cm incision of the skin was made on the left proximal hind limb at the level of the hip bone. The sciatic nerve was exposed at the sciatic notch, we transected and removed 1cm length of sciatic nerve for a complete irreversible neurotmesis model. In the group B, we performed sham operations (incision and exploration of the sciatic nerve only) at the same time. To avoid infection, 800000 units of penicillin (HenRui Co, China) were administered via intramuscular injection over the 7 days following operation. Ketoprofen (1.0mg/kg, once daily, (Xinan Pharmaceutical Factory, China) were subcutaneously injected for three days. All surgical procedures were performed by an author who had 10 years of experience with microsurgical procedures.

### MRI acquisition technique

MR studies was performed before nerve surgery and was repeated 1 day, 3 days, 5 days, 1week, 2 weeks, 3weeks 4 weeks, 6 weeks, 8 weeks, 10 weeks and 12 weeks after surgery. A venous indwelling needle (24 Gauge) was inserted into a lateral ear vein and secured for intravenous administration of anesthetics and MR contrast agent. All examinations were conducted on a 3 Tesla MR unit (Magnetom Trio Tim; Siemens, Erlangen, Germany). The measurements were performed with the rabbits under anesthesia and in a prone position with both hind limbs positioned in a dedicated eight-channel knee coil (Invivo, Gainesville, FL, USA). The MRI protocol included the following sequences: (1) Axial T1-weighted by turbo spin-echo (TSE) sequence images were obtained by using the following parameters: repetition time (TR), 824ms; echo time (TE), 22ms; matrix size, 320 × 320; field of view (FOV), 120 × 120mm; slice thickness, 3mm. (2) Axial T2-weighted images by TSE with fat suppression was obtained with the following parameters: TR, 4000; TE, 52; matrix size 320 × 320; FOV, 120 × 120mm; slice thickness, 3mm; (3) In the term of DCE imaging, for the baseline T1 mapping, unenhanced T1-weighted volume interpolated gradient echo (VIBE) images were acquired at each of the 3 flip angles using the following parameters: TR, 4.6ms; TE, 1.6ms; flip angle (FA), 2°, 8°, and 15°; FOV, 120× 120mm; matrix, 192 × 192; slice thickness, 3 mm. Then, DCE MR imaging using a radial three-dimensional VIBE with k-space-weighted image contrast reconstruction was performed. After 3 acquisitions, a bolus injection of 0.1 mmol/kg Gd-DTPA (gadopentetate dimeglumine, Magnevist, Bayer Schering, Berlin, Germany) was injected at a rate of 1 mL/s through the venous indwelling needle. Bolus injection was performed with a MR-compatible power injector (Spectris; Medrad, Pittsburgh, PA) followed by a 2-mL saline flush. The parameters were as follows: TR, 4.6ms; TE,1.6ms; FA, 11°; FOV, 120× 120mm; matrix, 192 × 192; slice thickness, 3mm; time resolution per measurement, 6 seconds; total scan duration, 10min.

### Image analysis

We subjectively classified signal intensity changes in the denervated gastrocnemius muscle into the following four grades: grade 0 (iso-signal intensity compared with that in the control group); grade 1 (slightly hyperintense); grade 2 (intermediate to high signal intensity); grade 3 (high signal intensity). Two authors (more than 10 years of experience with musculoskeletal MR imaging) evaluated the images in consensus.

The DCE-MRI data was post-processed by in-house software (OmniKinetics Version 2.0, GE Healthcare, China). The current tracer-kinetic modeling for quantitation of DCE images are based on a modified tofts model [22]. The model describes the tissue as a combination of a vascular compartment and an extracellular extravascular compartment, the following concentration-time equation was used:

$$C(t)=K^{trans}\;\int_{0}^{t}C_{p}(\tau )e^{k_{ep}(t-\tau )}d\tau +v_{p}C_{p}(t)$$ (1)

Where K^trans^ is the volume transfer constant between blood plasma and the extracellular extravascular space (EES); K_ep_ is the rate constant between the EES and blood plasma; V_p_ is blood plasma volume fraction; *C_p_*(*τ*) is the concentration-time curve in the arterial blood plasma. For quantitation of DCE images, the contrast concentration was semi-quantitated by using relative signal intensity enhancement ratio. For the purpose of the vascular input function (VIF), a region of interest (ROI) was placed in the popliteal artery, the mean size of ROIs was 4 mm^2^ (range, 3–5 mm2). Then the pixel-by-pixel plots of K^trans^, K_ep_ and V_p_ maps were automatically constructed by a Levenberg-Marquardt nonlinear least squares algorithm. ROIs were manually drawn on all imaging sections to acquire a volume of interest (VOI) of gastrocnemius muscle, and an effort was made to exclude fatty septa and vessels. Then, the mean K^trans^, K_ep_, and V_p_ values derived from Color-coded parametric maps were obtained. The mean VOIs of the gastrocnemius muscle were 4.54 ± 2.18 cm^3^. The above DCE parameters were measured by two independent observers (more than 10 years of experience with musculoskeletal MR imaging, respectively) who were blinded to the group allocations. The results acquired from two observers were averaged and used for analysis.

### EMG study

A Cadwell Sierra (Neuropack Four Mini; Nihon Kohden, Tokyo, Japan) computer-based EMG unit was used for electrodiagnostic studies, two rabbits randomly selected from each group were again anesthetized, and a conventional concentric needle electrode was placed in the left gastrocnemius muscle. We looked for abnormal spontaneous activity (positive sharp waves or fibrillation). The measurements were performed before nerve surgery and were repeated at 1 day, 3 days, 5 days, 1week, 2 weeks, 3weeks, 4 weeks, 6 weeks, 8 weeks, 10 weeks and 12 weeks after surgery by an author who had 10 years of experience with EMG.

### Statistical analysis

All statistical analyses were performed using SPSS 17.0 (SPSS, Inc., Chicago, IL,USA). Continuous variables were expressed as mean ± standard deviation (SD). Interobserver reliability of the measurements was assessed by using the intraclass correlation coefficient (ICC). The ICC was interpreted as follows: poor agreement (ICC = 0); minor agreement (ICC = 0–0.2); fair agreement (ICC = 0.21–0.40); moderate agreement (ICC = 0.41–0.60); good agreement (ICC = 0.61–0.8), perfect agreement (ICC = 0.81–1.00). K^trans^, K_ep_, and V_p_ values of specific acquisition points within groups were compared by using repeated-measures analysis of variance. Comparisons between the two groups at each acquisition point were performed by using one-way analysis of variance (ANOVA) test, statistical significances was considered when *P* value of less than 0.05.

## Results

### Changes in signal intensity

In the irreversible neurotmesis group (Group A), T2-weighted images with fat suppression showed slightly hyperintense in the target gastrocnemius muscle beginning 5 days after surgery, at 3 weeks, high signal intensity were observed. For all rabbits in this group, signal intensity remained high until 12-week follow up (Table 1).

**Table 1:** Classification of signal intensity changes for each injury group.

Follow-up interval	Group A (n = 12)				Group B (n = 12)			
	Grade 0	Grade 1	Grade 2	Grade 3	Grade 0	Grade 1	Grade 2	Grade 3
**Before**	12				12			
**1 d**	12				12			
**3 d**	12				12			
**5 d**	2	10			12			
**1 W**		12			12			
**2 W**		3	9		12			
**3 W**			1	11	12			
**4 W**				12	12			
**6 W**				12	12			
**8 W**				12	12			
**10 W**				12	12			
**12 W**				12	12			

W = week.

### Quantitative DCE MR imaging parameters

Kappa values (κ) of 0.823, 0.852, and 0.792 for K^trans^, K_ep_, and V_p_, respectively, show good interobserver agreement.

**Fig 1:**
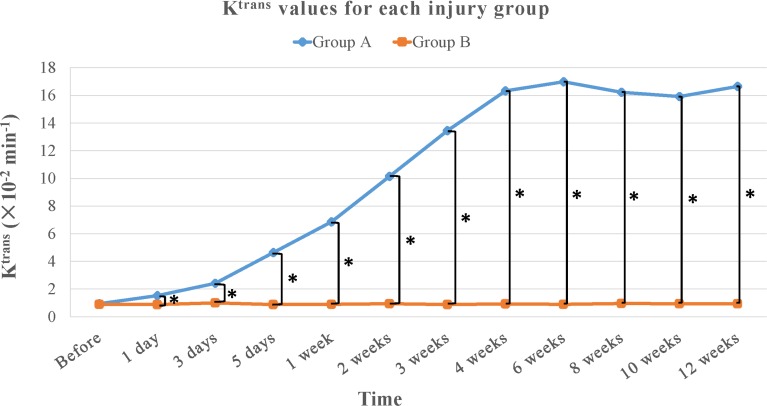
Graph shows the time course of K^trans^ values for each injury group. There were significant differences between irreversible neurotmesis group (group A, blue line) and control group (group B, orange line) after surgery (*P* < 0.05). _*_ = *P* < 0.05.

**Fig 2:**
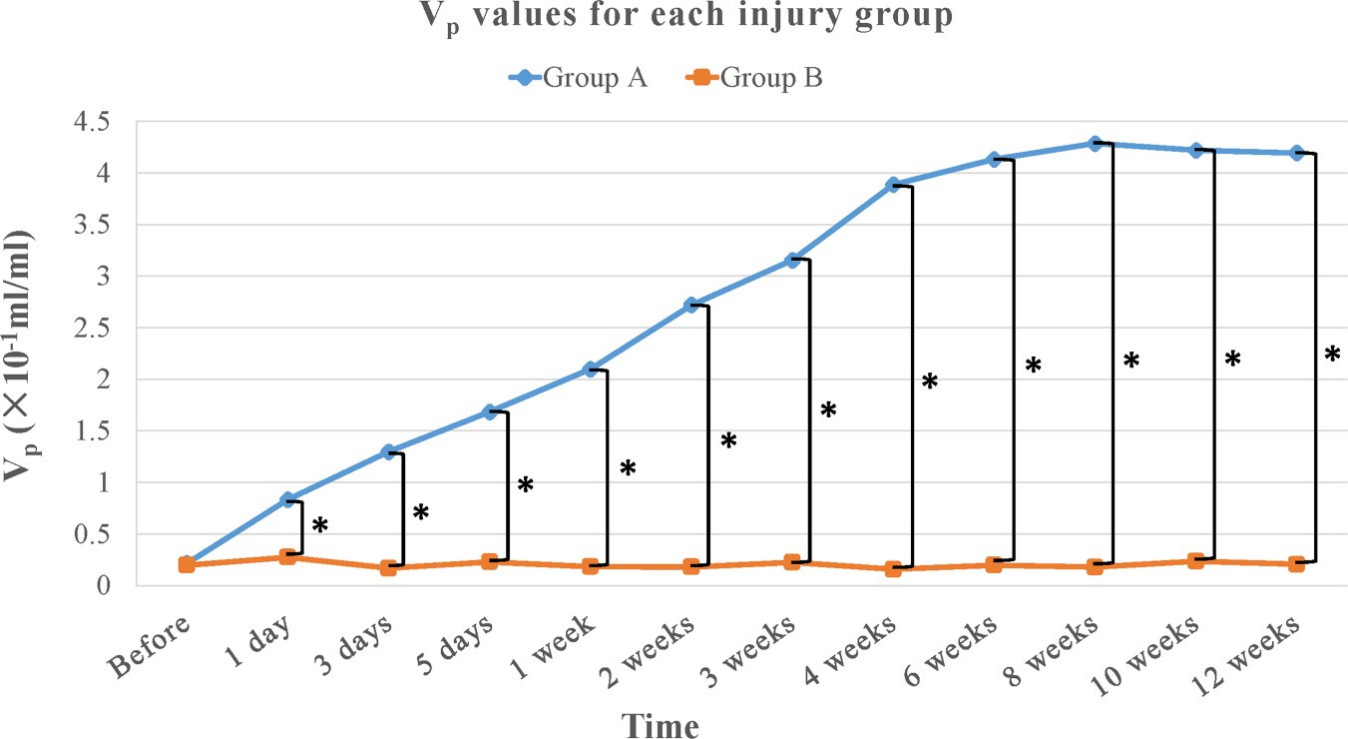
Graph shows the time course of V_p_ values for each injury group. There were significant differences between irreversible neurotmesis group (group A, blue line) and control group (group B, orange line) after surgery (*P* < 0.05). _*_ = *P* < 0.05.

**Fig 3:**
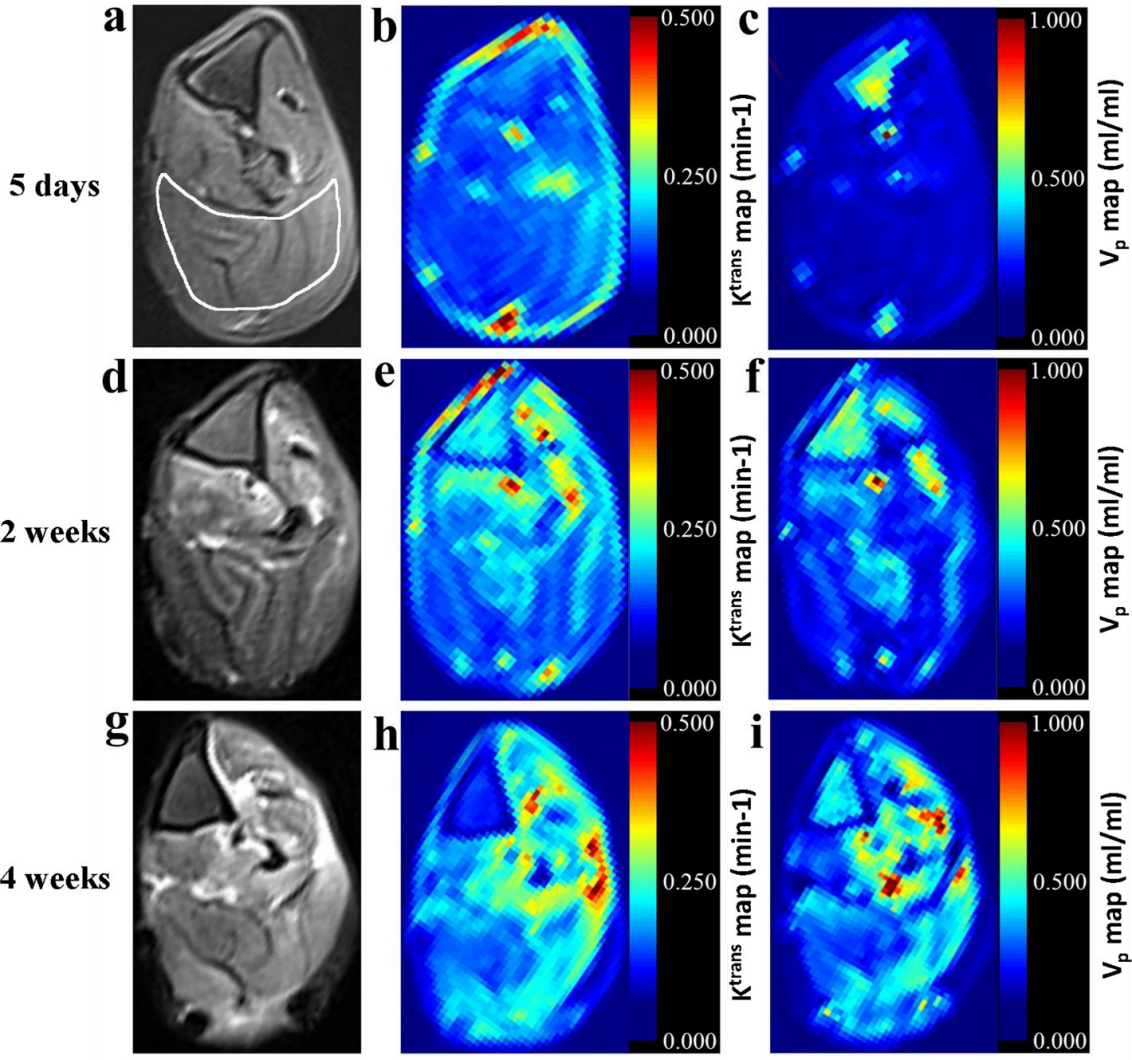
Axial fat-suppressed T2-weighted MR images of the left gastrocnemius muscle (outlined muscle in image) obtained with rabbits in the prone position. (a): At 5 days after surgery, image shows grade 1 signal intensity (ie, signal intensity was isointense or slightly hyperintense compared with that in the control group). (b, c): Color-coded parametric maps are derived from DCE MR imaging. Corresponding K^trans^ and V_p_ values are 4.513 ×10^−2^ min^-1^, 0.152ml/ml, respectively. (d): At 2 weeks after surgery, image shows grade 2 signal intensity (ie, intermediate to high signal intensity compared with that in the control group). (e, f): Color-coded parametric maps are derived from DCE-MR imaging. Corresponding K^trans^ and V_p_ values are 11.681 ×10^−2^ min^-1^, 0.272ml/ml, respectively. (g): At 4 weeks after surgery, image shows grade 3 signal intensity(ie, high signal intensity compared with that in the control group). (h, i): Color-coded parametric maps are derived from DCE MR imaging. Corresponding K^trans^ and V_p_ values are 16.203 ×10^−2^ min^-1^, 0.422ml/ml, respectively.

In the irreversible neurotmesis group (group A), K^trans^, V_p_ values increased gradually until 4 weeks after surgery and remained high (mean, approximately 15.391 ± 2.829 × 10^−2^ min^-1^ and 3.893 ± 0.890 × 10^−1^, respectively,) throughout the remainder of the study period (Tables 2 and 3, Figs 1, 2 and 3). In this group, we observed significant increases in both K^trans^ and V_p_ values compared with those in the control group during the period from1 day after surgery to 12 weeks after surgery (Tables 2 and 3, Figs 1, 2 and 3). K^trans^ values increased significantly during the periods from pre-operation to1 day after surgery (*P* = 0.029), from 1 day after surgery to 3 days after surgery (*P* < 0.001), from 3 days after surgery to 5 days after surgery (*P* < 0.001), from 5 days after surgery to 1 week after surgery (*P* < 0.001), from 1 week after surgery to 2 weeks after surgery (*P* < 0.001), from 2 weeks after surgery to 4 weeks after surgery (*P* < 0.001). V_p_ values increased significantly during the periods from pre-operation to 1 day after surgery (*P* < 0.001), from 1 day after surgery to 3 days after surgery (*P* = 0.001), from 3 days after surgery to 1 week after surgery (*P* < 0.001), from 1 week after surgery to 2 weeks after surgery (*P* = 0.007). From 2 weeks after surgery to 4 weeks after surgery (*P* = 0.001) However, K_ep_ values show no significant difference between the irreversible neurotmesis group and the control group.

**Table 2:** Mean K^trans^ values for each injury group.

Follow-up interval	Group A (×10^−2^ min^-1^)	Group B (×10^−2^ min^-1^)	*P*-Value
**Before**	0.904 ± 0.425	0.812 ± 0.424	0.600
**1 d**	1.444 ± 0.675	0.844 ± 0.294	0.010*
**3 d**	2.743 ± 0.806	0.921 ± 0.397	< 0.001*
**5 d**	4.540 ± 0.969	0.868 ± 0.428	< 0.001*
**1 W**	7.247 ± 0.999	0.874 ± 0.338	< 0.001*
**2 W**	10.859 ± 1.441	0.876 ± 0.468	< 0.001*
**3 W**	14.308 ± 3.722	0.881 ± 0.417	< 0.001*
**4 W**	15.391 ± 2.829	0.962 ± 0.282	< 0.001*
**6 W**	16.315 ± 3.672	0.975 ± 0.552	< 0.001*
**8 W**	17.858 ± 5.304	0.944 ± 0.479	< 0.001*
**10 W**	15.689 ± 5.086	0.929 ± 0.588	< 0.001*
**12 W**	16.628 ± 5.339	0.900 ± 0.426	< 0.001*

W = week. *P*-value represents the comparison results between group A and B by the one-way analysis of variance analyzation.

*P values indicate statistical significance.

**Table 3:** Mean V_p_ values for each injury group.

Follow-up interval	Group A(×10^-1^ml/ml)	Group B (×10^−1^ ml/ml)	*P*-Value
**Before**	0.218 ± 0.037	0.210 ± 0.031	0.594
**1 d**	0.834 ± 0.217	0.217 ± 0.085	< 0.001*
**3 d**	1.287 ± 0.299	0.182 ± 0.055	< 0.001*
**5 d**	1.646 ± 0.470	0.217 ± 0.087	< 0.001*
**1 W**	2.097 ± 0.435	0.187 ± 0.064	< 0.001*
**2 W**	2.717 ± 0.546	0.201 ± 0.113	< 0.001*
**3 W**	3.164 ± 1.221	0.225 ± 0.100	< 0.001*
**4 W**	3.893 ± 0.890	0.188 ± 0.082	< 0.001*
**6 W**	4.179 ± 0.967	0.194 ± 0.050	< 0.001*
**8 W**	4.253 ± 0.974	0.195 ± 0.056	< 0.001*
**10 W**	4.218 ± 1.120	0.236 ± 0.057	< 0.001*
**12 W**	4.197 ± 1.472	0.216 ± 0.088	< 0.001*

W = week. *P*-value represents the comparison results between group A and B by the one-way analysis of variance analyzation.

*P values indicate statistical significance.

### EMG study

In the irreversible axonotmesis group (group A), spontaneous activity was observed at 2-week follow-up; in the control group (group B). EMG was completely normal throughout the study period.

## Discussion

As in other studies, we demonstrate here that denervated skeletal muscle in the acute and subacute phase characteristically show high signal intensity on T2-weighted MR images [5, 6, 9]. However, to our knowledge, few studies available to date have used DCE MR imaging to evaluate denervated skeletal muscle. In this study, we evaluated the utility of DCE MR imaging for assessing denervated skeletal muscles in rabbits. Among the three different DCE MR imaging-derived parameters (K^trans^, K_ep_, and V_p_ values) used in this study, we found that compared to the uninjured nerves group (group A) showed a significant increase in K^trans^ and V_p_ values of denervated skeletal muscle after surgery.

For DCE MR imaging parameters, we found K^trans^ and V_p_ values increased significantly in the irreversible axonotmesis groups compared those in the control group during the stage of denervation. MR contrast agent Gd-DTPA does not enter the intracellular compartment and passes freely through the endothelial barrier with a rapid equilibrium between the interstitial and intravascular space [23]. Therefore, the increased K^trans^ and V_p_ values could be caused by a dilatation of the vascular bed, an enlargement of the extracellular space, or both. A previous study by Wessig et al found a significant increase of muscle capillaries at 2 days after denervation, and peak capillary enlargement was reached at 4 weeks follow up [24]. Another study by Eisenberg et al using radiolabeled microspheres in rats found a ten-fold increase in blood flow in denervated skeletal muscle. Several other experimental studies also have suggested that injury of sympathetic vasoconstriction leads to increased blood volume in denervated skeletal muscle [11, 25–27]. In the study by Yamabe et al and Holl et al, they demonstrated that extracellular fluid space significantly increased in the denervated skeletal muscle. Denervation causes increases in proteolysis [28], and the permeability muscle cell membrane [29], and decrease in protein synthesis and glycolysis [30]. Also, loss of neurotrophic factor [31], disuse atrophy caused by immobilization, and functional disability of Na,K-ATPase may occur[32]. All of these changes could increase the extracellular water volume. In the present study, we found increase of K^trans^ and V_p_ values in denervated skeletal muscle as early as 1 day after surgery, i.e., about 2 weeks before the appearance of EMG abnormities. Compared with signal intensity changes, the K^trans^ and V_p_ values obtained from DCE MR imaging further narrowed this diagnostic gap.

K_ep_ values represents the flux rate constant between the EES and blood plasma, in the present study, there was no significant differences in K_ep_ values between the irreversible axonotmesis group and the control group. In our opinion, it may be due to contrast enhanced pattern. In our study, we found that both the denervated skeletal muscle and normal skeletal muscle in the rabbits showed persistent pattern (type-I) time-intensity curve (TIC). During the scan time, the contrast in the skeletal muscle constantly full-filled into the EES. Considered that K_ep_ is the flux rate constant from EES to blood plasma, it was not surprising to find that there was no significant difference in K_ep_ values between these two groups.

MRI changes in denervated skeletal muscles were first described by Polak et al in 1988[7], Fifteen days after transection of the sciatic nerves in rats, prolongation of T2 TR was observed in the denervated skeletal muscles of the lower legs. Thereafter, some other studies also demonstrated that the denervated skeletal muscles presented with high signal intensity on T2-weighted MR images. This phenomenon of high signal intensity on T2-weighted image in denervated skeletal muscles might correlate with an increase in extracellular fluid [6, 7, 9].

In the present study, denervated skeletal muscles showed increased signal intensity that started 5 days after surgery, and presented continuously high signal intensity from 3 weeks after surgery. The time course of signal intensity changes seen on T2-weighted MR images of denervated skeletal muscle in our study was inconsistent with those in previous reports. The study by Holl et al demonstrated that increased signal intensity in denervated skeletal muscle occurred as early as 1 day after sciatic nerve axotomy [9], whereas in another study by Küllmer et al, increased signal intensity on T2-weighted images in denervated skeletal muscle has been shown to occur as early as 3 weeks after suprascapular nerve transection [33]. This discrepant results might be due to different nerve injury pattern [8], denervation develops earlier when the nerve was cut near the muscle. Long peripheral nerve stump allows a longer release of neurotrophic and protective factors than short stump [7].

In our study, the EMG findings after experimental denervation were consistent with those in the literature [6, 8], about 2–3 weeks after denervation, the first changes (spontaneous activity) were found. When compared with EMG findings, the observed MR signal changes were an early phenomenon occurred about one week before the earliest EMG abnormalities after experimental denervation in rabbits.

Our study had some limitations. First, the results of kinetic parameters may vary when using different post-processing model, and it may also be influenced by following factors: MR scanning parameters, vascular input function, methods for T1 measurement, and injection rate of contrast agent. However, this is common issue which was reported by many previous researches, and we will not address it in this study. Second, in our study, we used a 6-second temporal resolution, relatively lower than those of established articles [34, 35]. The low temporal resolution may result in an underestimate of the VIF, thus leading to overestimate V_p_ and K^trans^ values of skeletal muscle. This was a technical limitation because our current MR sequences did not support faster scanning for so large imaging coverage. Afterall, the quantitative results here reflect significant differences between injured and control groups, which demonstrate that DCE is a reliable tool to determine early and time-course hemodynamic changes in denervated skeletal muscles in a rabbit model. Third, the present study focused only on complete nerve transection. Less severe nerve damage and regeneration was not considered in this study. However, an article by Yamable et al. demonstrated that T2 ratios depended on the degree of nerve injury, the more severe the nerve damage, the higher signal intensity on T2-weighted images [8]. Forth, we consider the results to be consistent with changes at MR imaging in human muscle. Further clinical study will be needed in human subject.

## Conclusion

In summary, K^trans^ and V_p_ values obtained from DCE MR imaging parameters changes occur as early as 1 day after denervation. Increased K^trans^ and V_p_ values is an early phenomenon markedly preceding not only EMG abnormalities but also signal intensity changes after nerve transection. DCE MR imaging hold the promise of an early and sensitive diagnosis of denervated skeletal muscle.

## Supporting information

S1 Tables Data of the dynamic contrast-enhanced magnetic resonance imaging in denervated skeletal muscle: experimental study in rabbits. (PDF)

S1 Appendix The ARRIVE guidelines checklist (Animal research: Reporting in vivo experiment). (PDF)
