# Supplementary material for: Dynamic contrast-enhanced magnetic resonance imaging in denervated skeletal muscle: Experimental study in rabbits
Source: PLoS One. 2019 Apr 5;14(4):e0215069. doi: 10.1371/journal.pone.0215069 (PMC6450635; doi:10.1371/journal.pone.0215069)
Supplement: S1 Tables — (PDF) [file pone.0215069.s001.pdf]

Dynamic contrast-enhanced magnetic resonance imaging in denervated skeletal muscle: experimental study in rabbits

Liang Qi, Lei Xu, Wen-Tao Wang, Yu-Dong Zhang, Rui Zhang, Yue-Fen Zou, Hai-Bin Shi

**Ktrans values for each injury group(×10-2 min-1）**												
**Follow-up interval**	**before surgery**	**1d**	**3d**	**5d**	**1wk**	**2wk**	**3wk**	**4wk**	**6wk**	**8wk**	**10wk**	**12wk**
**Group A-rabbit 1**	1.192	1.258	3.533	4.276	6.859	13.670	16.299	12.747	19.073	13.107	20.534	13.175
**Group A-rabbit 2**	1.032	0.576	2.734	5.468	7.686	9.716	9.615	17.973	14.164	14.155	14.627	8.489
**Group A-rabbit 3**	0.630	2.583	3.324	5.379	6.052	9.358	14.004	18.645	20.310	11.713	13.419	13.222
**Group A-rabbit 4**	1.376	2.179	3.304	4.605	7.618	11.994	20.915	17.070	8.934	19.118	14.061	27.826
**Group A-rabbit 5**	0.609	1.859	1.898	6.112	7.731	12.489	15.677	18.948	18.514	13.914	11.080	16.751
**Group A-rabbit 6**	0.917	0.372	4.292	4.760	7.357	9.892	17.622	17.694	17.220	29.133	25.947	20.271
**Group A-rabbit 7**	0.313	1.292	2.092	4.465	6.234	10.905	8.435	11.341	18.769	20.221	19.497	20.797
**Group A-rabbit 8**	1.547	0.812	2.777	5.157	8.193	8.506	12.196	17.057	17.449	20.943	16.321	16.484
**Group A-rabbit 9**	1.422	1.343	1.931	3.231	8.914	10.145	10.961	15.330	17.905	12.911	7.119	17.655
**Group A-rabbit 10**	0.940	2.238	2.243	2.881	8.274	11.284	15.884	11.827	14.229	23.583	10.748	11.213
**Group A-rabbit 11**	0.577	1.401	3.159	4.734	6.220	11.353	12.211	12.983	10.136	20.816	19.284	12.510
**Group A-rabbit 12**	0.298	1.412	1.626	3.407	5.831	11.001	17.884	13.084	19.078	14.684	15.629	21.138
**Group B-rabbit 1**	1.009	0.391	1.378	0.452	0.448	1.196	0.723	0.649	0.894	0.240	0.428	1.532
**Group B-rabbit 2**	0.739	0.893	0.940	0.589	0.624	0.680	1.008	1.392	1.339	0.359	0.815	0.740
**Group B-rabbit 3**	1.422	0.876	0.414	1.533	1.402	1.455	1.515	0.704	0.459	1.514	1.793	0.646
**Group B-rabbit 4**	0.256	0.897	0.355	0.260	0.663	0.842	1.289	0.740	0.210	1.188	1.230	1.303
**Group B-rabbit 5**	1.718	0.841	0.877	1.404	0.731	0.100	0.351	1.083	0.290	0.312	0.344	1.361
**Group B-rabbit 6**	0.355	1.361	0.992	0.923	1.242	1.273	1.281	1.197	1.004	1.267	0.060	0.113
**Group B-rabbit 7**	0.452	0.621	1.297	0.906	1.078	1.074	0.336	0.602	0.939	1.609	1.033	0.994
**Group B-rabbit 8**	0.897	0.969	1.305	0.613	0.357	1.618	0.761	1.097	1.580	1.042	0.506	1.332
**Group B-rabbit 9**	0.744	0.932	0.589	0.891	1.002	0.579	0.998	0.924	1.446	0.565	1.646	0.991
**Group B-rabbit 10**	0.846	0.362	1.082	0.338	1.096	0.922	0.939	0.930	1.663	1.386	1.806	0.609
**Group B-rabbit 11**	0.779	0.742	1.417	1.341	1.204	0.313	1.169	1.439	1.583	0.920	0.927	0.714
**Group B-rabbit 12**	0.530	1.243	0.402	1.165	0.643	0.461	0.206	0.789	0.295	0.929	0.560	0.467

Vp values for each injury group												
**Follow-up interval**	**before surgery**	**1d**	**3d**	**5d**	**1wk**	**2wk**	**3wk**	**4wk**	**6wk**	**8wk**	**10wk**	**12wk**
**Group A-rabbit 1**	0.291	1.287	1.525	1.507	2.586	2.793	2.792	4.546	4.748	5.769	3.795	5.271
**Group A-rabbit 2**	0.201	0.481	1.344	1.644	2.560	2.453	2.157	2.873	3.830	5.076	2.450	6.373
**Group A-rabbit 3**	0.256	0.903	0.562	1.563	1.187	2.191	1.450	2.849	6.490	4.706	6.230	4.232
**Group A-rabbit 4**	0.240	0.672	1.277	0.582	2.862	2.352	6.513	3.896	3.912	3.529	4.072	4.145
**Group A-rabbit 5**	0.237	0.587	1.684	2.392	2.153	3.319	3.654	2.656	4.085	3.236	3.900	4.339
**Group A-rabbit 6**	0.185	0.827	1.038	1.468	2.135	2.608	2.967	3.687	4.928	3.106	3.026	1.845
**Group A-rabbit 7**	0.183	0.788	1.419	2.001	2.023	3.291	1.995	4.585	3.297	3.588	4.668	5.397
**Group A-rabbit 8**	0.218	0.923	1.083	1.649	2.208	1.927	3.245	4.845	3.556	4.736	3.257	6.197
**Group A-rabbit 9**	0.170	0.700	1.037	1.441	2.003	2.495	2.779	3.732	4.431	6.028	3.507	1.337
**Group A-rabbit 10**	0.258	0.741	1.604	2.482	1.673	3.603	3.582	5.842	4.949	3.526	4.272	4.136
**Group A-rabbit 11**	0.176	0.969	1.439	1.524	2.113	3.462	2.928	3.641	2.884	3.287	5.851	3.782
**Group A-rabbit 12**	0.196	1.128	1.436	1.503	1.660	2.105	3.906	3.560	3.037	4.446	5.585	3.315
**Group B-rabbit 1**	0.261	0.141	0.165	0.386	0.219	0.004	0.168	0.295	0.130	0.232	0.254	0.032
**Group B-rabbit 2**	0.241	0.287	0.195	0.197	0.283	0.127	0.125	0.147	0.161	0.228	0.224	0.123
**Group B-rabbit 3**	0.180	0.336	0.241	0.124	0.144	0.230	0.057	0.200	0.220	0.147	0.147	0.239
**Group B-rabbit 4**	0.250	0.089	0.187	0.169	0.171	0.281	0.247	0.021	0.209	0.180	0.218	0.256
**Group B-rabbit 5**	0.187	0.178	0.082	0.151	0.244	0.285	0.318	0.229	0.265	0.265	0.236	0.206
**Group B-rabbit 6**	0.219	0.277	0.136	0.265	0.206	0.188	0.241	0.145	0.166	0.184	0.375	0.158
**Group B-rabbit 7**	0.234	0.112	0.207	0.307	0.221	0.161	0.215	0.285	0.230	0.078	0.184	0.335
**Group B-rabbit 8**	0.183	0.360	0.196	0.063	0.249	0.101	0.175	0.165	0.146	0.207	0.213	0.324
**Group B-rabbit 9**	0.193	0.218	0.217	0.262	0.207	0.468	0.478	0.131	0.196	0.176	0.172	0.232
**Group B-rabbit 10**	0.212	0.227	0.093	0.289	0.060	0.264	0.248	0.247	0.112	0.132	0.252	0.179
**Group B-rabbit 11**	0.202	0.132	0.281	0.150	0.143	0.113	0.193	0.100	0.220	0.213	0.273	0.334
**Group B-rabbit 12**	0.155	0.249	0.185	0.240	0.092	0.189	0.232	0.292	0.275	0.294	0.284	0.169

Kep values for each injury group(×10-2 min-1）												
**Follow-up interval**	**before surgery**	**1d**	**3d**	**5d**	**1wk**	**2wk**	**3wk**	**4wk**	**6wk**	**8wk**	**10wk**	**12wk**
**Group A-rabbit 1**	0.457	0.332	0.201	0.410	0.354	0.390	0.656	0.468	0.442	0.293	0.361	0.478
**Group A-rabbit 2**	0.366	0.336	0.245	0.271	0.341	0.037	0.336	0.338	0.191	0.214	0.324	0.491
**Group A-rabbit 3**	0.522	0.271	0.169	0.307	0.403	0.355	0.384	0.350	0.156	0.276	0.154	0.361
**Group A-rabbit 4**	0.376	0.319	0.521	0.431	0.578	0.385	0.108	0.410	0.042	0.202	0.310	0.262
**Group A-rabbit 5**	0.263	0.481	0.391	0.257	0.361	0.428	0.261	0.369	0.260	0.321	0.171	0.244
**Group A-rabbit 6**	0.204	0.163	0.380	0.122	0.317	0.432	0.297	0.178	0.306	0.274	0.351	0.140
**Group A-rabbit 7**	0.287	0.237	0.270	0.202	0.229	0.445	0.197	0.245	0.286	0.320	0.312	0.261
**Group A-rabbit 8**	0.324	0.449	0.108	0.333	0.138	0.251	0.269	0.405	0.315	0.221	0.330	0.295
**Group A-rabbit 9**	0.432	0.150	0.164	0.306	0.254	0.223	0.384	0.394	0.456	0.369	0.263	0.447
**Group A-rabbit 10**	0.270	0.270	0.350	0.339	0.140	0.373	0.189	0.268	0.251	0.370	0.202	0.247
**Group A-rabbit 11**	0.355	0.360	0.289	0.378	0.526	0.271	0.329	0.340	0.392	0.169	0.034	0.252
**Group A-rabbit 12**	0.210	0.133	0.549	0.320	0.231	0.378	0.161	0.161	0.392	0.260	0.400	0.386
**Group B-rabbit 1**	0.383	0.256	0.468	0.375	0.198	0.291	0.287	0.161	0.385	0.681	0.190	0.279
**Group B-rabbit 2**	0.438	0.281	0.168	0.295	0.310	0.407	0.334	0.384	0.185	0.413	0.375	0.256
**Group B-rabbit 3**	0.408	0.418	0.184	0.394	0.331	0.515	0.326	0.335	0.227	0.297	0.326	0.088
**Group B-rabbit 4**	0.174	0.412	0.205	0.332	0.413	0.141	0.325	0.306	0.420	0.354	0.334	0.422
**Group B-rabbit 5**	0.332	0.261	0.163	0.322	0.284	0.283	0.303	0.395	0.350	0.222	0.342	0.661
**Group B-rabbit 6**	0.313	0.485	0.362	0.231	0.114	0.404	0.307	0.365	0.143	0.444	0.318	0.436
**Group B-rabbit 7**	0.229	0.655	0.290	0.392	0.137	0.197	0.008	0.263	0.190	0.220	0.195	0.365
**Group B-rabbit 8**	0.489	0.440	0.541	0.310	0.402	0.222	0.250	0.278	0.341	0.209	0.410	0.191
**Group B-rabbit 9**	0.367	0.197	0.322	0.506	0.491	0.176	0.253	0.378	0.303	0.278	0.490	0.398
**Group B-rabbit 10**	0.375	0.263	0.401	0.491	0.297	0.293	0.435	0.242	0.208	0.317	0.309	0.240
**Group B-rabbit 11**	0.199	0.311	0.411	0.334	0.384	0.266	0.114	0.192	0.283	0.384	0.259	0.265
**Group B-rabbit 12**	0.329	0.379	0.104	0.438	0.162	0.199	0.444	0.193	0.129	0.420	0.616	0.416
